# Should AI allocate livers for transplant? Public attitudes and ethical considerations

**DOI:** 10.1186/s12910-023-00983-0

**Published:** 2023-11-27

**Authors:** Max Drezga-Kleiminger, Joanna Demaree-Cotton, Julian Koplin, Julian Savulescu, Dominic Wilkinson

**Affiliations:** 1 Faculty of Medicine, Nursing and Health Sciences, Monash University, Melbourne, Australia; 2 Oxford Uehiro Centre for Practical Ethics, Faculty of Philosophy, University of Oxford, Oxford, OX1 2JD UK; 3 Monash Bioethics Centre, Monash University, Melbourne, Australia; 4 Murdoch Children’s Research Institute, Melbourne, Australia; 5 Centre for Biomedical Ethics, Yong Loo Lin School of Medicine, National University of Singapore, Singapore, Singapore; 6 John Radcliffe Hospital, Oxford, UK

**Keywords:** Artificial intelligence, Bioethics, Organ allocation, Transplantation, Algorithms, Public attitudes, Resource allocation, Machine learning

## Abstract

**Background:**

Allocation of scarce organs for transplantation is ethically challenging. Artificial intelligence (AI) has been proposed to assist in liver allocation, however the ethics of this remains unexplored and the view of the public unknown. The aim of this paper was to assess public attitudes on whether AI should be used in liver allocation and how it should be implemented.

**Methods:**

We first introduce some potential ethical issues concerning AI in liver allocation, before analysing a pilot survey including online responses from 172 UK laypeople, recruited through Prolific Academic.

**Findings:**

Most participants found AI in liver allocation acceptable (69.2%) and would not be less likely to donate their organs if AI was used in allocation (72.7%). Respondents thought AI was more likely to be consistent and less biased compared to humans, although were concerned about the “dehumanisation of healthcare” and whether AI could consider important nuances in allocation decisions. Participants valued accuracy, impartiality, and consistency in a decision-maker, more than interpretability and empathy. Respondents were split on whether AI should be trained on previous decisions or programmed with specific objectives. Whether allocation decisions were made by transplant committee or AI, participants valued consideration of urgency, survival likelihood, life years gained, age, future medication compliance, quality of life, future alcohol use and past alcohol use. On the other hand, the majority thought the following factors were not relevant to prioritisation: past crime, future crime, future societal contribution, social disadvantage, and gender.

**Conclusions:**

There are good reasons to use AI in liver allocation, and our sample of participants appeared to support its use. If confirmed, this support would give democratic legitimacy to the use of AI in this context and reduce the risk that donation rates could be affected negatively. Our findings on specific ethical concerns also identify potential expectations and reservations laypeople have regarding AI in this area, which can inform how AI in liver allocation could be best implemented.

**Supplementary Information:**

The online version contains supplementary material available at 10.1186/s12910-023-00983-0.

## Background

Livers are scarce, and transplantation is the only treatment for those with end-stage hepatic failure [1, 2]. Therefore, difficult decisions must be made in allocating donor livers. These involve complex predictions of donor organ and recipient interactions, and competing ethical values, including utility, urgency, justice, and responsibility [3].

Artificial Intelligence (AI) has been proposed to assist in decision-making in many areas of medicine [4]. While definitions vary, medical AI can be defined as *“an information system capable of considering data and making clinical or patient care decisions commonly associated with a human”*, which can use rule-based and/or non-rule based algorithms [5]. The former have specific rules set by experts and have been used in medicine since the 1970s [6]. Non-rule-based or machine learning algorithms are more complex and “learn” from vast amounts of data to detect patterns and make predictions [7, 8]. AI can now diagnose certain medical conditions equally (or more) accurately than specialist doctors [9–11]. AI has also been proposed to assist in resource allocation, for example in ICU prognosis and organ allocation [12, 13].

Algorithms have assisted in liver allocation for some time and have been increasing in complexity [14]. The Model for End-Stage Liver Disease (MELD) score is a basic algorithm which uses the results of three blood tests to predict how urgently a patient requires a liver (more accurately than clinicians) [15, 16]. Transplant units worldwide have used MELD scores to rank patients on waiting lists since the early 2000s [14].

However, using the MELD score (and its modifications) to rank patients is simplistic and there are concerns regarding its accuracy [17, 18]. This means human input by clinicians and transplant committees is required to consider additional factors which might be relevant (such as expected outcome) and to adjust decisions for certain circumstances. This potentially risks inconsistency, the influence of cognitive biases and deliberate manipulation [19, 20].

Furthermore, there has been a shift to incorporate further consideration of predicted outcomes into allocation, to maximise the utility of what is a scarce resource – particularly given a push to transplant poorer quality organs to increase supply [14]. In the UK, livers are first offered to those on the “super-urgent” list [21]. If there are no patients on this list, patients are ranked by their Transplant Benefit Score, an algorithm which uses 21 recipient and 7 donor characteristics to predict a patient’s “survival benefit” from a transplant [14]. This therefore incorporates both predicted urgency and outcome into allocation policy. However, independent simulations suggest that this algorithm may still be too simplistic to accurately predict survival benefit for certain subgroups of waiting list patients (i.e., those with hepatocellular carcinoma) [22].To consider additional factors and improve access to certain groups, the US has also started implementing a new algorithmic organ allocation system where specific factors are chosen and given a weighting, and patients are then ranked based on those factors [23].^1^ The effects of this system on patient outcomes and fairness of distribution remain to be seen.

The development of liver allocation policies from MELD score (and its predecessors) to newer algorithms has been driven by a push to improve outcomes (for example via more accurate predictions of survival), and the desire to balance several complex factors in a way that is consistent. However, given the number of variables involved, the complexity of these allocation systems has the potential to increase even further [19, 25]. Many more complex algorithmic and AI models have been proposed or could be adapted for use in liver allocation (Table 1). These largely aim to improve on making predictions, for example of urgency, post-transplant survival or years of life to gain from a transplant. Given the potential beneficial effect on patient outcomes that may arise from more accurate predictions, and the trend towards using more complex allocation systems, we must explore the ethical implications of using algorithms and AI in this context.

**Table 1:** AI predictive model proposals

AI model prediction	Example proposals	General relevant findings
Pre-transplant mortality (urgency)	Cucchetti, 2007; Bertsimas, 2019	AI models more accurate than MELD score [26, 27].
Post-transplant survival	Cruz-Ramírez, 2013; Briceño, 2014; Lau, 2017; Matis, 1995; Ayllón, 2018; Ershoff, 2020; Haydon, 2005; Hoot, 2005; Khosravi, 2015; Dorado-Moreno, 2017; Zhang, 2012	Predict survival at 30 days, 3 months, 1–5 years post-transplant [2, 28–37]. Most more accurate than current scoring systems.
Survival benefit ** (e.g., life years gained)**	Schaubel, 2009; Dancs, 2022	Predict survival benefit (using pre-and post-transplant survival predictions) [38, 39].
QALYs	Santos, 2020	Predict 30-day quality-adjusted survival (in critically ill cancer patients) [40]. None in organ allocation: feasible area of future research.

The ethical status of using AI for medical purposes remains controversial. Some have argued that, as well as improving accuracy, the use of algorithms will also lead to more impartial, consistent, and efficient decision-making [4, 5, 41–44]. On the other hand, there is ethical concern about AI bias [4, 45, 46] and that some (non-rule-based) types of AI are a “black box”, i.e., the decision-making process is uninterpretable to the user [45, 47, 48]. Broader concerns, such as the loss of important human elements or nuance in healthcare decision-making are also common [42, 45, 49, 50]. Specific concerns relating to AI in liver allocation have not been systematically explored, nor are the views of the public about this development known. However, a qualitative study of US transplant centre clinicians’ attitudes towards AI in liver allocation identified several key ethical themes in this context, relating to explainability, transparency, fairness, and trustworthiness [44]. Overall, the study found that clinicians were “cautiously optimistic” about the use of AI in this space.

On the other hand, studies have repeatedly shown that when all things are equal, laypeople prefer humans making medical or ethical decisions compared to computers [51–54]. However, it is unclear whether preference for human decision-making over AI extends to organ allocation. A study of attitudes to AI in kidney organ allocation found that preferences for AI or human decision-makers were relatively split [55]. No studies have thus far assessed layperson attitudes towards AI in liver allocation.

## PART 1: why might we use algorithms and AI in liver allocation?

AI is increasingly used in medical domains – for example, to assist in diagnosing cancers using medical imaging [56]. Liver allocation, however, deals with a decision that is not (merely) medical (as in the case of medical diagnosis), but rather *ethical*: to whom should a scarce, life-saving resource be allocated?

**Fig. 1:**
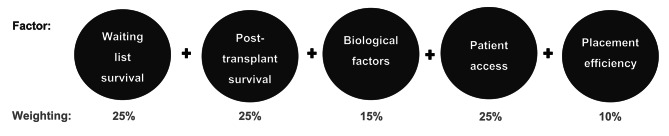
US Organ Procurement and Transplantation Network lung allocation system [24]. Each individual factor is given a specific weighting, which is used to give patients on the waiting list a ranking

There are challenges to using algorithms and AI in ethical decision-making [45, 47]. A main obstacle is achieving sufficient agreement on normative values to distil them into programmable variables. It may be obvious how to program AI to detect lung cancers on chest X-rays, but it would clearly be more difficult to create AI to decide when life support should be discontinued in intensive care. However, much depends on the approach we use to programming, and on the ethical values that are relevant to the specific context. We will introduce liver allocation as an area where there are some pro tanto reasons for using AI (although we will fall short of conclusively claiming that it should be used here). Firstly, there are top-down and bottom-up approaches to programming AI in liver allocation. Top-down would involve either single or multiple objectives being explicitly programmed. This could look similar to the recently approved US organ allocation policy, with specific weighted factors used to rank patients (Fig. 1) [24]. On the one hand, this sort of programming requires explicit endorsement of specific ethical values, which may be more difficult than native human ethical decision-making processes; health professionals in the emergency department may intuitively include factors in triaging patients, based on predicted urgency, outcome etc., but are not necessarily asked to write down factors and weightings used. However, in organ allocation, policies are generally transparent and publicly available anyway, even if humans make the decisions [57, 58]. If we can publish specific objectives in guidelines, programming specific objectives into AI is also feasible.

One potential concern with top-down AI, is that it may be too inflexible to account for nuances required for some types of decisions. For example, consider the difficulty in programming top-down AI with sufficient discrete variables to make end of life decisions for patients in intensive care, where (amongst other factors) judgments of future quality of life must be made. However, as mentioned, liver allocation is already largely algorithm-based [14] and factors which are currently already considered (such as urgency and predicted survival) may be more easily quantified. Given this, it might only be a small step to using top-down AI for this purpose, and an algorithmic process would allow for more consistent decision-making. Relative to traditional allocation algorithms, increasing the complexity of AI algorithms is also likely to mean more factors can be considered and decisions are more accurate [27, 32]. Accuracy may refer to making relevant predictions, for example of urgency and chance of survival, but also may refer to the accuracy by which an AI system applies programmed rules to a decision. For example, a transplant guideline may specify that livers should be primarily allocated based on urgency, but human decision-makers may erroneously give more weight to predicted survival when making a decision. AI programmed to give 70% weight to urgency and 30% weight to predicted survival is less likely to make this error. If AI can make predictions and weigh factors with a higher degree of accuracy and greater consistency than human decision-makers, then this provides one reason in favour of using it to use it to allocate livers. (This is a contingent empirical claim; if AI were less accurate/consistent than humans, we should not use it).

Alternately, AI could be programmed bottom-up, for example by training a machine learning algorithm on previous allocation decisions made by humans. Assuming the AI is accurate, this would result in similar decisions to those made currently or in the past, but with some of the advantages of speed, efficiency and decreased moral burden on those making decisions. Decisions would also potentially be more consistent, in that patients with identical features should yield the same decision. Another advantage of this method is that it bypasses the difficult question of how to explicitly weight multiple factors, as the AI would learn to do this based on how humans have in the past. Of course, this risks systematising and amplifying biases in the way decisions are currently made (although if trained on the verdicts of multiple transplant committees, using AI could dampen the effects of some biases which may affect any individual committee [59]). Implementing any changes to the current system would also be difficult — for example if we decided to improve access to transplants for certain groups (as is the case in the new US policy), some top-down element would be required to make this possible.

Therefore, some have suggested that bottom-up AI should not be used as a decision-replacement, though it may have benefits as a decision-aid [59]. For example, if the AI recommends a course of action different to the transplant committee, and specifies the values and weights it used to make that recommendation (i.e., is not a black-box), this may assist human decision-makers to examine their own, comparatively opaque reasoning process.

There are several potential reasons to deploy AI in liver allocation. Both methods of programming AI in this context have advantages over human decision-making, but whether these outweigh the real concerns is unclear. One significant concern is whether AI in liver allocation would be accepted by the public. This is a particularly critical consideration as public approval of allocation policy may affect organ donation rates [20].

This pilot study aimed firstly, to indicate whether there is some public support for the use of AI in a high-stakes resource allocation context. Secondly, we aimed to identify respondents’ views on key issues regarding the design and implementation of AI in liver allocation. This may provide insight into whether the use of AI in this context is likely to be democratically legitimate. Additionally, these results may help identify ethical issues which require further exploration.

## PART 2: public attitudes

### Methods

Participants were recruited through the online platform Prolific Academic. Respondents were at least 18 years of age, fluent in English, based in the UK and had a minimum Prolific approval rate of 96%. The sample was gender balanced. The survey was created using Qualtrics XM and pre-tested on colleagues and a smaller Prolific sample.

A sample size of 200 was chosen based on time and resource constraints. Post-hoc power analysis suggested that a sample size of 172 (accounting for excluded responses) gave us 90% power to detect medium effect sizes (d = 0.5) in differences between conditions at a significance level of 0.05, and > 99% power to detect within-participant differences between questions. Precision analysis, based on a UK population size of 67 million [60], suggested that this would give a 7% margin of error at a 95% confidence level.

Questions were largely scenario-based, and responses recorded on 7-point Likert scales. Statements and questions in each section were presented in a randomised order (full survey in Appendix A).

#### Attitudes towards use of AI in liver allocation

##### AI acceptability

Participants were given explanations of AI and different areas of medicine, before being asked the extent to which they found the use of AI acceptable in those areas. They were also asked the extent to which they agreed with the statement: “If AI were used in liver allocation, I would be less likely to donate my organs”. To assess ethical concerns that could drive these acceptability ratings, participants were asked how they felt about AI compared to humans regarding four key ethical issues identified from previous literature [45]: consideration of decision-making nuances, dehumanisation of healthcare, bias, and consistency.

##### Decision-maker characteristics

Participants were told that decision-makers could possess different characteristics: interpretability, empathy, accuracy, consistency, and impartiality (which were chosen based on discussions amongst authors and are consistent with themes present in the relevant literature [44, 61, 62]). These terms were explained, and respondents were asked to divide a total of 100 points amongst these to indicate their relative importance.

#### Preferences for design of AI in liver allocation

To identify views about how AI might be used, participants were asked whether AI should be trained on previous human decisions (bottom-up) or programmed with specific factors (top-down).

Then, to assess whether the type of decision-maker would affect views on which factors should be incorporated into allocation, participants were randomised (within the survey platform) to either a “transplant committee” or “AI” condition. Both groups then were given 13 prompts regarding factors (identified in part from previous literature [3, 13]) that could be relevant to liver allocation. Participants rated the extent to which these factors should affect priority – when used by either a transplant committee or an AI decision-maker. These factors were: urgency, survival likelihood, life years gained, age, future medication compliance, quality of life, past alcohol use, future alcohol use, past crime, future crime, future societal contribution, socioeconomic status, and gender.

Finally, respondents were asked whether an AI decision should be overridable by a transplant committee. (Analysis of additional questions can be found in Appendix D.)

#### Statistical analysis

Statistical analysis was conducted using IBM SPSS Statistics. We used descriptive statistics to measure the frequency of various responses. For comparisons, Likert scales were assigned number values from 1 to 7 (for example where 1 = totally unacceptable and 7 = perfectly acceptable) and t-tests were performed to compare mean scores. Multiple linear regression was used to assess whether certain responses predicted views about the overall acceptability of AI for liver allocation. A *p*-value of < 0.05 was considered significant.

The project was reviewed and approved by the University of Oxford Central University Research Ethics Committee (R80692/RE003) as well as Monash University Ethics Committee (project number 34,555).

### Results

Two hundred participants completed the survey. Twenty-eight were excluded for failing at least one of two attention checks (N = 172). The median age category was 35–44, 93.6% of respondents had completed high school or higher education, 88.4% identified as white, and 62.8% identified as having no religion (full demographics in Appendix B).

#### Attitudes towards use of AI in liver allocation

##### AI acceptability

**Fig. 2:**
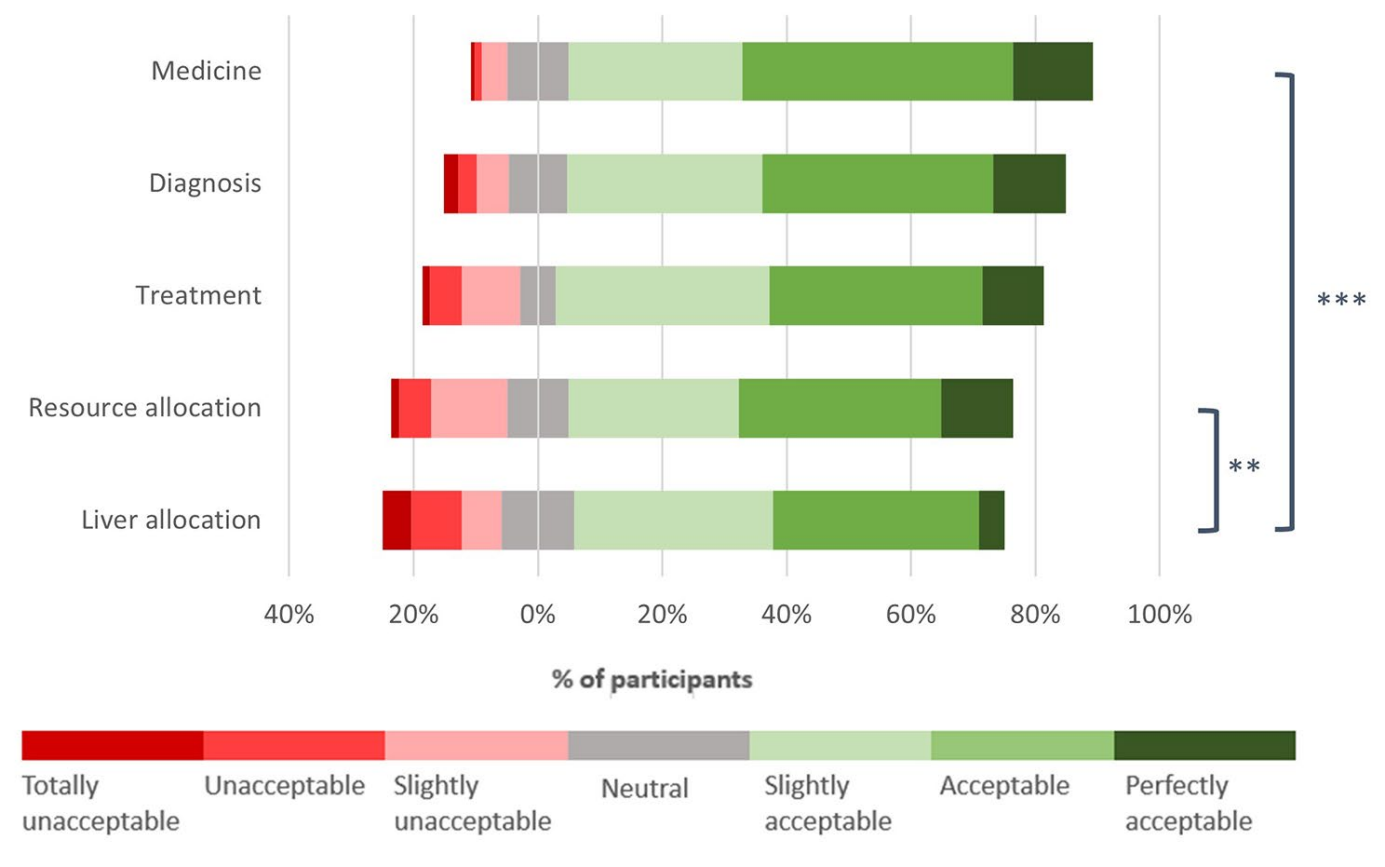
Public attitudes towards AI in medicine. Bars represent how acceptable participants found the use of AI in different areas of medicine. **Participants found AI in liver allocation (M = 3.26, SD = 1.51) less acceptable compared to resource allocation (M = 2.99, SD = 1.43), t(171) = 3.19, p = .002. *** Participants found AI in liver allocation (M = 3.26, SD = 1.51) less acceptable compared to medicine (M = 2.55, SD = 1.10), t(171) = 7.26, p < .001. *Where 1 = Perfectly acceptable, 7 = totally unacceptable*

A majority found the use of AI acceptable in all areas of medicine that were asked about (Fig. 2). Of all respondents, 84.3% found the use of AI in medicine acceptable (slightly acceptable, acceptable, or perfectly acceptable) compared to 69.2% in liver allocation. On average, participants found AI in liver allocation less acceptable than AI in medicine or resource allocation generally, although this effect was marginal. Behavioural questions revealed similarly positive views: 72.7% of respondents disagreed with the statement that if AI were used in liver allocation, they would be less likely to donate their organs, while 10.5% agreed and 16.8% felt neutrally (Appendix C).

We then analysed attitudes towards AI that could drive acceptability ratings. Of all respondents, 82.0% thought that AI was likely to make less biased decisions than humans and 89% thought that AI would be more consistent than humans. However, 73.3% thought that AI was less likely to take into consideration nuances of individual liver allocation situations compared to humans, and 61.6% agreed with a statement that AI would lead to the dehumanisation of healthcare (Appendix D). Acceptability ratings were predicted by the extent to which AI is perceived as leading to the dehumanisation of healthcare (*B* = 0.294, *p* < .001), the extent to which it is perceived as more or less likely to consider the individual nuances of individual situations (β=-0.168, *p* = .021), and the extent to which AI is perceived to be more or less biased than humans (β = 0.157, *p* = .032). Perceptions of consistency did not significantly predict acceptability ratings (β=-0.097, *p* = .185). Further analysis can be found in Appendix D.

##### Decision-maker characteristics

**Fig. 3:**
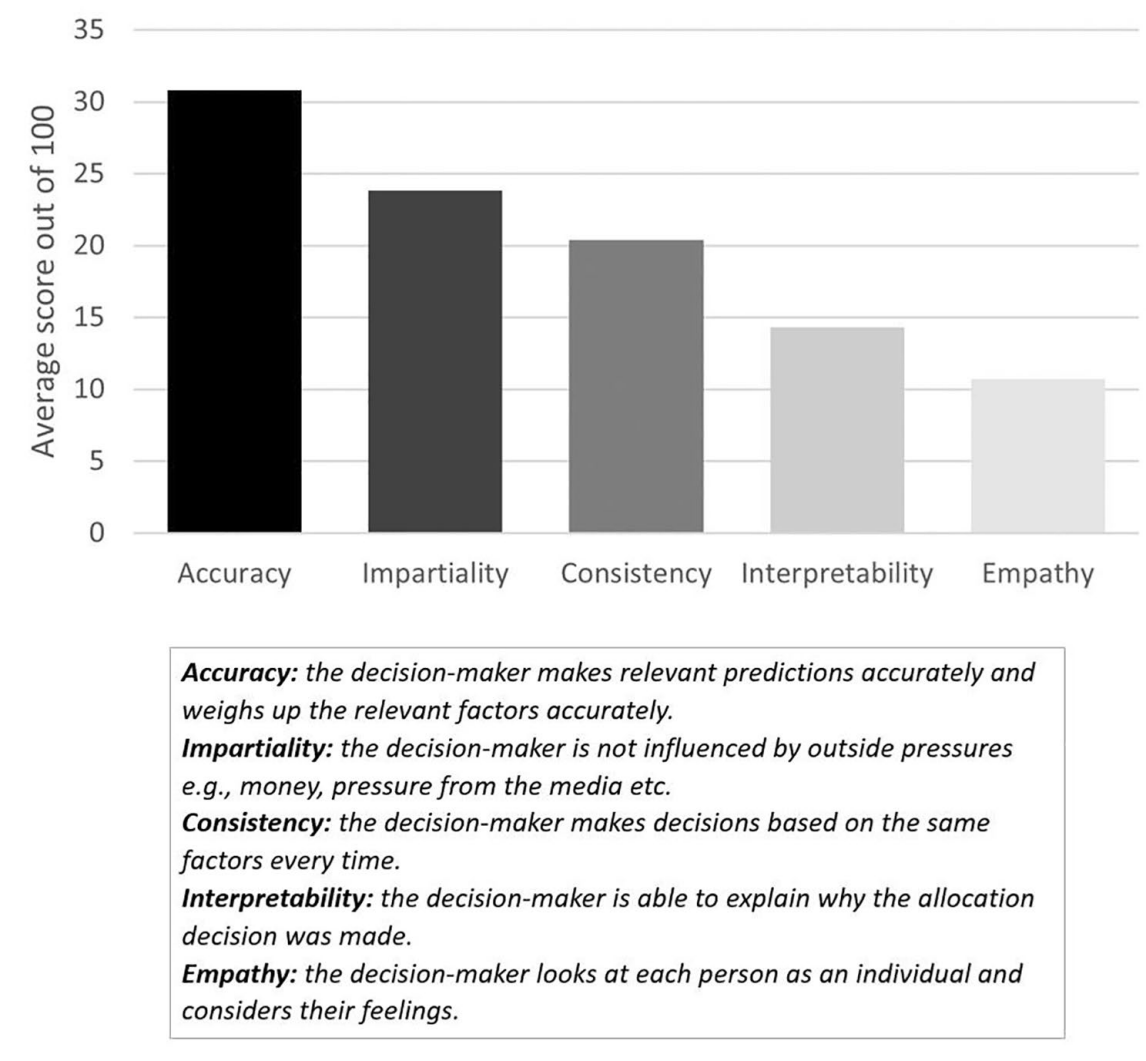
Public attitudes towards the importance of characteristics of decision-makers. The bars depict the average score (out of 100) that each characteristic received. Definitions that participants received are also depicted

Participants found accuracy the most important characteristic for a liver allocation decision-maker, followed by impartiality, consistency, interpretability, and empathy (Fig. 3). This same order was found by ranking the characteristics by highest average score or the number of participants choosing characteristics as their first or last ranked (Appendix E).

#### Preferences for design of AI in liver allocation

Of all respondents, 40.7% preferred AI to learn from previous human decisions, 34.9% preferred AI to be programmed with specific objectives, and 24.4% were neutral towards this.

**Fig. 4:**
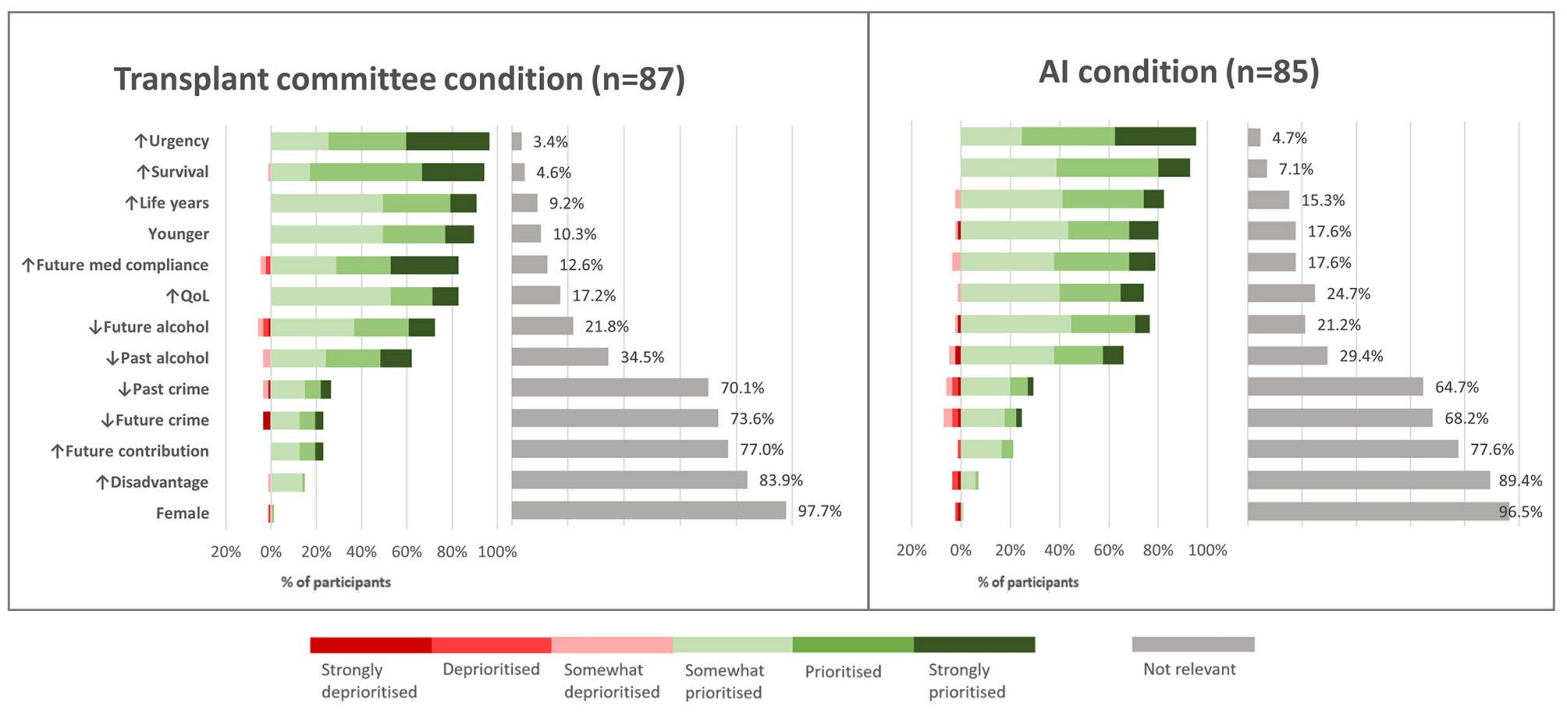
Public attitudes to liver allocation priority factors (transplant committee and AI groups). Results are shown from the transplant committee condition on the left and the AI condition on the right. Each bar represents a factor which could be used in liver allocation. Green bars represent participants who thought that patients should be prioritised based on that factor (and red bars represent those who thought they should be deprioritised). Separate grey bars represent those who thought that factor was not relevant to liver allocation

Most participants thought that the following characteristics should give patients priority for liver allocation: greater urgency, survival likelihood, life years gained, being younger, future medication compliance, quality of life, lower future alcohol use and lower previous alcohol use (Fig. 4). On the other hand, the majority thought the following factors were not relevant to prioritisation: past crime, future crime, future societal contribution, disadvantage, and female gender. Whether the decision was made by AI or transplant committee had very little impact on participant views of which factors should be included in allocation decisions. (Fig. 4). See Appendix F for further analysis.

Of all respondents, 87.2% agreed that liver allocation AI should be overridable by a transplant committee (either strongly agreed, agreed, or somewhat agreed).

### Discussion

#### Attitudes towards use of AI in liver allocation

##### AI acceptability

Several findings in our study appear to indicate support for the use of AI in liver allocation. Firstly, respondents found the use of AI acceptable in all areas of medicine that were asked about, including liver allocation. Some studies have indicated that the public generally prefer human decision-makers to AI in medical and ethical decision-making, [51–54] even if there may not be an explicit reason for this [63]. Therefore, to avoid responses skewed unfairly against AI, we simplified the question and asked how acceptable people find AI without a human comparator. This may have resulted in more positive responses towards AI. Secondly, we found that most participants stated they would be just as likely to donate organs if AI were used in allocation. However, while only 10% of participants indicated that they were less likely to donate, a 10% reduction in donation rates would be a serious negative consequence. It would be important to confirm in other studies, whether those who indicated a negative response would donate in the absence of AI, i.e., to determine whether there would be a true reduction in donation rates.

A previous study has proposed “uniqueness neglect” – i.e., the idea that reducing patients to a number misses something important about their situation – as a reason for public aversion to medical AI [50]. Our study likewise indicates that this may be relevant, as most participants thought that AI would be less likely to consider nuances of allocation decisions, and, although the effect was not very strong, this predicted a slightly lower acceptance response for AI.^2^ Most participants also agreed that AI would lead to the dehumanisation of healthcare, and the more they thought this, the more unacceptable they found AI for liver allocation. In fact, this was the strongest predictor of acceptability ratings of all the ethical concerns measured. This is consistent with a previous study [49]. These findings are useful as they offer an avenue towards practical solutions: for example, Longoni et al. found that explaining to patients the ways in which AI does actually consider individual factors appears to decrease aversion to AI [50]. Similarly, explaining to patients the extent that humans are still involved in the creation of algorithms may also alleviate some concern regarding dehumanisation.

Unsurprisingly, participants expected AI to be a more consistent decision-maker, although this did not appear to have an impact on overall acceptability ratings. While concerns about biased AI are common in the literature, they appear to be less significant in the public eye. One previous study found participants to be more concerned with human biases [49], which appears to complement our finding that participants thought that AI would be likely to make less biased decisions, compared to humans. This may reflect that participants may be more familiar with human biases that could affect healthcare professionals, compared to statistical biases that could affect AI decisions. Alternately, the public may also be more accepting of discrimination by algorithms rather than humans [64]. As familiarity should not guide whether discrimination or bias is problematic, some caution is warranted in attributing normative weight to these views.

##### Decision-maker characteristics

Participant ranking of characteristics for allocation decisions lends additional support to the use of AI in liver allocation. Respondents valued (in order of importance): accuracy, impartiality, consistency, interpretability, and empathy in those making decisions about liver allocation. AI is likely to be more accurate than humans in this context and most of our participants indicated that they expected AI to make less biased and more consistent decisions. Therefore, this appears to implicitly favour AI as the decision-maker. However, we acknowledge that these five characteristics do not necessarily capture all the potential characteristics of an allocation decision-maker - a different or expanded set of variables might have yielded a different conclusion.

Many studies have highlighted the importance of AI accuracy to the public [50, 51, 55], and it is perhaps unsurprising that this was seen as the most important factor for decisions. Interpretability was the second-least important characteristic, which is interesting, since interpretability is commonly discussed in AI ethics. There are often considered to be epistemic and ethical reasons why AI should be interpretable [12, 47]. Some have argued that black-box AI is inherently problematic because transparent explanations of decisions are indispensable for a fair decision-making process, and promote trust and acceptance - an (at least partially) contingent empirical claim [12, 48, 65]. However, previous work has similarly indicated that the public value accuracy more than interpretability in AI-assisted medical resource allocation [61, 62]. Empathy was rated as the least important characteristic by our participants. This appears to be consistent with some clinicians’ views that AI’s lack of emotion may be beneficial for the liver transplant evaluation process [44].

#### Preferences for design of AI in liver allocation

To our knowledge, this is the first study to attempt to gather public opinion comparing bottom-up and top-down AI used in ethical decision-making [66]. While AI based on previous human decisions (i.e., bottom-up AI) was slightly preferred, responses were split. This question is quite complex and therefore these results are perhaps not unexpected.

Prioritisation factors identified by participants match up well with those which are currently used in liver allocation policies across the world: for example urgency, survival, life-years gained, young age [14, 57].^3^ Interestingly, participants indicated that the same ethical factors were relevant to liver allocation, regardless of whether top-down AI or a transplant committee made the predictions and weighted the factors. This might be practically useful information as this suggests that we could program top-down AI with the same values that we currently use for liver allocation guidelines. Secondly, some relevant factors (e.g., predicted life-years gained) involve calculations with hundreds of relevant variables and may be difficult for humans to make reliably [19]. If that factor is deemed relevant, this might support the use of AI in this area.

This was a small study and results may not extrapolate to the whole population (nor to other populations), although demographics were roughly comparable to the UK [60] and modest online convenience samples have been shown to yield similar results to representative sampling [68]. Prolific is a validated platform for recruiting survey participants, the population is limited to those who have access to the internet and have time for online surveys. AI and liver allocation are also complex: we attempted to provide sufficient information about AI without overloading readers, however, this is likely to be a survey of relatively intuitive responses rather than considered opinions. Further work assessing clinician perceptions of AI in liver allocation would also provide valuable information for the implementation of this technology (for example, it may indicate whether clinicians are likely to follow the allocation indicated by an AI). One recent study suggests US transplant centre clinicians are relatively optimistic about this use of AI [44].

## Conclusion

This paper opens the door to further discussion and investigation in a relatively unexplored area of AI ethics. Complex algorithms are becoming more common in resource allocation, but the benefits of these must be further appraised and traded off with the potential concerns. One prior concern was whether the public would accept or reject this use of this technology. Our sample of UK participants appeared to support the use of AI in liver allocation and the majority were no less likely to donate their organs if AI was used. Interestingly, participants found accuracy to be a more important characteristic in allocation, compared to interpretability and empathy, which also may favour AI. These findings, if confirmed, would give democratic legitimacy to the use of AI in liver allocation, and mitigate concerns that donation rates could be adversely affected.

Additionally, our participants were open to either top-down programming with explicit values embedded into AI, or bottom-up programming utilising machine learning from human decision making. Regardless of whether livers were allocated by a transplant committee or AI, participants thought urgency, survival life years gained, and age were the most relevant factors to be considered, which is consistent with previous ethical analysis and current allocation policies. Further work is required to assess how these should be traded off, as well as to appraise some of the more contentious factors. Our findings on specific ethical concerns, also identify avenues for improving the way AI could be implemented.

The field of AI is moving rapidly. Since the time of this survey, large language models such as ChatGPT have gained enormous popularity and have become the topic of much debate. These models could feasibly be asked to choose between transplant patients using the methods described in this paper: by giving a set of ethical values and weightings (top-down) or by asking the model to summarise previous decisions (bottom-up). Clearly, this would require more rigorous testing, however it is apparent that we can no longer speak of this technology in hypothetical terms: AI *could* allocate livers. Although we have not concluded that AI *should* be used in high-stakes decision-making areas such as liver allocation, our study may help inform debate on this important question.

### Electronic supplementary material

Below is the link to the electronic supplementary material.

Supplementary Material 1

Supplementary Material 2

Supplementary Material 3

Supplementary Material 4

Supplementary Material 5

Supplementary Material 6

## Data Availability

The datasets used during the current study are available from the corresponding author on reasonable request.

## Footnotes

1 This has already been implemented for lung allocation in March 2023, but is still in development for liver allocation [24].

2 There may be two concerns here: the idea that missed nuances may affect decision accuracy, and a perceived lack of patient-centredness.

3 Other factors which were also deemed relevant such as quality of life are also consistent with other proposed approaches to scarce resource allocation [67].
