# Supplementary material for: Should AI allocate livers for transplant? Public attitudes and ethical considerations
Source: BMC Med Ethics. 2023 Nov 27;24:102. doi: 10.1186/s12910-023-00983-0 (PMC10683249; doi:10.1186/s12910-023-00983-0)
Supplement: Supplementary file 1 — Supplementary Material 1 [file 12910_2023_983_MOESM1_ESM.pdf]

APPENDIX A

Full Survey:

Start of Block: Acceptability

Artificial intelligence (AI) is increasingly used to help in making medical and public health decisions.
For this survey we are using the term “medical AI” to refer to:
“an information system capable of considering data and making clinical or patient care decisions”.

AI systems can process large amounts of data, “learn” from it and use that information to make predictions and decisions. For example, studies have shown that medical AI can detect skin cancers more accurately than many skin specialists.

AI may be able to consider more complex medical factors and make more objective and consistent decisions than humans. However, like humans, medical AI isn’t perfect at making decisions. For example, AI can make mistakes if there are issues in how it is programmed or the data it is based on is flawed.

We will now ask you some questions about your opinion on the use of AI in medicine. For each of these questions please assume that it is technically possible to use AI and that there is evidence that it is at least as accurate as humans.

The use of AI in medicine is:

- ○ Perfectly acceptable
- ○ Acceptable
- ○ Slightly acceptable
- ○ Neutral
- ○ Slightly unacceptable
- ○ Unacceptable
- ○ Totally unacceptable

In medicine, diagnosing a medical condition involves considering symptoms, the results of a physical examination and test results to reach a likely diagnosis. AI could be used to process this information to diagnose patients with medical conditions.
The use of AI to diagnose patients with medical conditions is:

- ○ Perfectly acceptable
- ○ Acceptable
- ○ Slightly acceptable
- ○ Neutral
- ○ Slightly unacceptable
- ○ Unacceptable
- ○ Totally unacceptable

In medicine, making treatment decisions involves weighing up factors such as how well a treatment works and what side effects the treatment has. AI could be used to process this information to decide what treatment is

appropriate for a patient.
The use of AI to make medical treatment decisions is:

- Perfectly acceptable
- Acceptable
- Slightly acceptable
- Neutral
- Slightly unacceptable
- Unacceptable
- Totally unacceptable

In the healthcare system, resources such as hospital beds, complex equipment and medical treatments are sometimes limited. AI could be used to process this information to decide who should receive these resources.
The use of AI to allocate scarce medical resources is:

- Perfectly acceptable
- Acceptable
- Slightly acceptable
- Neutral
- Slightly unacceptable
- Unacceptable
- Totally unacceptable

Liver transplantation is an effective treatment for many liver diseases, however there are not enough livers to go to everyone who needs one. Some people die waiting for a liver. Deciding who should receive a liver is a difficult task, which involves balancing medical and ethical factors. AI could be used to process this information to decide who should receive a liver.

The use of AI to allocate livers is:

- Perfectly acceptable
- Acceptable
- Slightly acceptable
- Neutral
- Slightly unacceptable
- Unacceptable
- Totally unacceptable

Liver transplantation is an effective treatment for many liver diseases, however there are not enough livers to go to everyone who needs one. Some people die waiting for a liver. Deciding who should receive a liver is a difficult task, which involves balancing medical and ethical factors. AI could be used to process this information to decide who should receive a liver.

Do you agree or disagree with the following statement:
"If AI were used in liver allocation, I would be less likely to donate my organs"

- ○ Strongly disagree
- ○ Disagree
- ○ Somewhat disagree
- ○ Neither agree nor disagree
- ○ Somewhat agree
- ○ Agree
- ○ Strongly agree

End of Block: Acceptability

Start of Block: Block 2 (Transplant committee condition)

Please imagine that a liver unexpectedly becomes available for transplantation. The liver would be a match for several current patients who have severe liver failure. All the patients are currently very unwell and there is a risk they will die in the coming weeks without a transplant. Whoever receives the liver will need to take several medicines a day to stop their body 'rejecting' the transplant.

There is a need to decide which patient should receive the transplant.

Transplant committees are a group of people whose job it is to make decisions about how to allocate organs. They often include health professionals such as doctors and nurses, but also non-medical people. The committee would be told to consider one or more factors in their decisions.

We are going to ask you for your views on how transplant committees might determine which patient should receive a liver.
We will give you statements about certain factors and asking whether this means a patient should be:
Deprioritised: this should move the patient lower on the waiting list
Not relevant: transplant committees should not consider this factor
Prioritised: this should move the patient higher on the waiting list

When a transplant committee allocates a liver, patients who are predicted to require a liver more urgently should be:

- Strongly deprioritised
- Deprioritised
- Somewhat deprioritised
- Not relevant
- Somewhat prioritised
- Prioritised
- Strongly prioritised
-

The rest of the statements in this block all have these 7 response options

When a transplant committee allocates a liver, patients with a higher predicted chance of survival should be:

When a transplant committee allocates a liver, patients with more predicted years of life to gain should be:

When a transplant committee allocates a liver, patients with more predicted quality of life to gain should be:

When a transplant committee allocates a liver, patients whose liver disease was not caused by alcohol use should be:

When a transplant committee allocates a liver, patients who are predicted to be more likely to take their anti-rejection medication should be:

When a transplant committee allocates a liver, patients are sick. Please select "prioritised" to indicate that you are still paying attention. This should be:

When a transplant committee allocates a liver, patients who are predicted to be less likely to drink alcohol in the future should be:

When a transplant committee allocates a liver, patients who are predicted to be less likely to commit a future crime should be:

When a transplant committee allocates a liver, patients who are predicted to be more likely to contribute to society should be:

When a transplant committee allocates a liver, patients from a disadvantaged background should be:

When a transplant committee allocates a liver, younger patients should be:

When a transplant committee allocates a liver, patients with less of a criminal record should be:

When a transplant committee allocates a liver, female patients should be:

End of Block: Block 2 (transplant committee condition)

Start of Block: Block 2 (AI condition)

Please imagine that a liver unexpectedly becomes available for transplantation. The liver would be a match for several current patients who have severe liver failure. All the patients are currently very unwell and there is a risk they will die in the coming weeks without a transplant. Whoever receives the liver will need to take several medicines a day to stop their body ‘rejecting’ the transplant.

There is a need to decide which patient should receive the transplant.

Artificial intelligence (AI) might be used to allocate livers, by being programmed with one or more factors that should be considered.

We are going to ask you for your views on how AI might determine which patient should receive a liver.
We will give you statements about certain factors and asking whether this means a patient should be:
Deprioritised: the patient should be lower on the waiting list
Not relevant: AI should not be programmed with this factor
Prioritised: this patient should be higher on the waiting list

Please assume there is evidence that the AI is at least as accurate as humans.

When AI is used to allocate a liver, patients who are predicted to require a liver more urgently should be:

- ○ Strongly deprioritised
- ○ Deprioritised
- ○ Somewhat deprioritised
- ○ Not relevant
- ○ Somewhat prioritised
- ○ Prioritised
- ○ Strongly prioritised

The rest of the statements in this block all have these 7 response options

When AI is used to allocate a liver, patients with a higher predicted chance of survival should be:

When AI is used to allocate a liver, patients with more predicted years of life to gain should be:

When AI is used to allocate a liver, patients with more predicted quality of life to gain should be:

When AI is used to allocate a liver, patients whose liver disease was not caused by alcohol use should be:

When AI is used to allocate a liver, patients who are predicted to be more likely to take their anti-rejection medication should be:

When a transplant committee allocates a liver, patients are sick. Please select "prioritised" to indicate that you are still paying attention. This should be:

When AI is used to allocate a liver, patients who are predicted to be less likely to drink alcohol in the future should be:

When AI is used to allocate a liver, patients who are predicted to be less likely to commit a future crime should be:

When AI is used to allocate a liver, patients who are predicted to be more likely to contribute to society should be:

When AI is used to allocate a liver, patients from a disadvantaged background should be:

When AI is used to allocate a liver, younger patients should be:

When AI is used to allocate a liver, patients with less of a criminal record should be:

When AI is used to allocate a liver, female patients should be:

End of Block: Block 2 (AI condition)

Start of Block: Block 3 – natural condition

Rejection is where a patient's immune system attacks a newly transplanted liver, which may result in the liver failing. **If a patient's immune system is more primed to attack a new liver, this is more likely.**

Imagine Patient A and Patient B are both on the liver transplant waiting list and a liver becomes available.

Based on a genetic test, an AI algorithm predicts that **patient A's immune system is more primed to attack the liver**, and is therefore twice as likely to have rejection and the liver to fail within 6 months, compared to patient B. The AI prediction is known to be right 90% of the time.

Indicate whether you agree or disagree with the following statement:
"Given the AI prediction, patient B should be prioritised over patient A."

- ○ Strongly disagree
- ○ Disagree
- ○ Somewhat disagree
- ○ Neither agree nor disagree
- ○ Somewhat agree
- ○ Agree
- ○ Strongly agree

End of Block: Block 3 – natural condition

Start of Block: Block 3 – behavioural condition

Rejection is where a patient's immune system attacks a newly transplanted liver, which may result in the liver failing. **If a patient doesn't strictly take certain anti-rejection medications after transplantation, this is more likely.**

Imagine Patient A and Patient B are both on the liver transplant waiting list and a liver becomes available.

Based on a genetic test, an AI algorithm predicts that **patient A is less likely to take their anti-rejection medication** and is therefore twice as likely to have rejection and the liver to fail within 6 months, compared to patient B. The AI prediction is known to be right 90% of the time.

Please indicate whether you agree or disagree with the following statement:
"Given the AI prediction, patient B should be prioritised over patient A."

- ○ Strongly disagree
- ○ Disagree
- ○ Somewhat disagree
- ○ Neither agree nor disagree
- ○ Somewhat agree
- ○ Agree
- ○ Strongly agree

End of Block: Block 3 – behavioural condition

Start of Block: Block 4: objective vs human-proxy

Artificial intelligence (AI) can be programmed with specific objectives, which could be the basis for allocating livers. Examples of these objectives may be: how urgently a patient requires a liver, how many years of life they are likely to gain from a transplant or other factors mentioned in previous sections.

Alternatively, AI could learn from previous allocation decisions made by liver transplant committees. The AI would analyse a large database of previous decisions and then uses what it has learnt about how humans make decisions to allocate organs.

Should AI be programmed with specific objectives or learn from previous human decisions?

- ○ Strongly prefer specific objectives
- ○ Prefer specific objectives
- ○ Somewhat prefer specific objectives
- ○ Neutral
- ○ Somewhat prefer previous human decisions
- ○ Prefer previous human decisions
- ○ Strongly prefer previous human decisions

End of Block: Block 4: objective vs human-proxy

Start of Block: Block 5: Overridability

Both AI and humans can make mistakes. AI is expected to become more accurate than humans in making predictions. Humans may be able take into account factors that have not been programmed into the AI.

Imagine there are clear criteria for liver allocation. Using these criteria an AI system indicates that patient A should receive a liver. The transplant committee disagrees with the decision and thinks patient B should receive the liver.

Please indicate whether you agree or disagree with the following statement:
"The transplant committee should be able to override the AI and allocate the liver to patient B instead."

- ○ Strongly disagree
- ○ Disagree
- ○ Somewhat disagree
- ○ Neither agree nor disagree
- ○ Somewhat agree
- ○ Agree
- ○ Strongly agree

End of Block: Block 5: Overridability

Start of Block: Block 6: Characteristics

Please imagine that a policy is agreed upon for liver allocation. The allocation decision would be made by a "decision-maker": either a group of humans (a transplant committee) or an AI system.

Different decision-makers may have different characteristics in regards to:
Interpretability: the decision-maker is able to explain why the allocation decision was made.
Empathy: the decision-maker looks at each person as an individual and considers their feelings.
Accuracy: the decision-maker makes relevant predictions accurately and weighs up the relevant factors accurately.
Consistency: the decision-maker makes decisions based on the same factors every time.
Impartiality: the decision-maker is not influenced by outside pressures e.g., money, pressure from the media etc.

How important are each of these characteristics for an AI or human making liver allocation decisions?
Using the sliding scales, please give each characteristic a score from 0 (NOT AT ALL IMPORTANT) to 100 (MOST IMPORTANT) as they relate to a decision-maker making liver allocation decisions.
You only have a maximum of 100 points to allocate across all 5 characteristics.

_______ Accuracy
_______ Interpretability
_______ Consistency
_______ Empathy
_______ Impartiality

End of Block: Block 6: Characteristics

Start of Block: Ethical issues

We will now ask you a series of other questions about the use of AI in liver allocation.

Do you think AI is more or less likely to consider nuances of individual liver allocation situations compared to humans?

- Much less likely
- Less likely
- Somewhat less likely
- Equally likely
- Somewhat more likely
- More likely
- Much more likely

Humans may make biased decisions by unfairly favouring certain patients, which may happen on purpose or without them even realising. AI may make biased decisions when the data it is trained on unfairly favours certain patients.
Do you think AI is likely to make more or less biased decisions compared to humans?

- Much less biased
- Less biased
- Somewhat less biased
- Neither more or less biased
- Somewhat more biased
- More biased
- Much more biased

Please indicate to what extent you agree or disagree with the following statement:
"AI will lead to the “dehumanisation of healthcare”: there will be less human interaction as part of healthcare.”

- Strongly disagree
- Disagree
- Somewhat disagree
- Neither agree nor disagree
- Somewhat agree
- Agree
- Strongly Agree

Do you think AI is more or less likely to make mistakes? If you are reading this, please select "somewhat more likely" to indicate you are still paying attention.

- Much less likely
- Less likely
- Somewhat less likely
- Equally likely
- Somewhat more likely
- More likely
- Much more likely

Do you think AI is more or less likely to make consistent decisions compared to humans?

- Much less consistent
- Less consistent
- Somewhat less consistent
- Equally Consistent
- Somewhat more likely
- More likely
- Much more likely

A liver is allocated by an AI system and a patient on the waiting list passes away. The parents of this patient believe their daughter should have received the liver instead of the patient who did.
Who should be held responsible for decisions made by an AI in liver allocation?

- The AI system itself
- The transplant committee/hospital using the AI
- The person/people who programmed the AI
- A combination of other options
- Nobody
- Unsure

End of Block: Ethical issues

Start of Block: Block 8: Utilitarian scale

We will now ask some other questions about your moral views.
Please indicate how much you agree or disagree with the following statements:

If the only way to save another person’s life during an emergency is to sacrifice one’s own leg, then one is morally required to make this sacrifice.

- Strongly disagree
- Disagree
- Somewhat disagree
- Neither agree nor disagree
- Somewhat agree
- Agree
- Strongly agree

The rest of the statements in this block all have these 7 response options

From a moral point of view, we should feel obliged to give one of our kidneys to a person with kidney failure since we do not need two kidneys to survive, but really only one to be healthy.

From a moral perspective, people should care about the well-being of all human beings on the planet equally; they should not favor the well-being of people who are especially close to them either physically or emotionally.

It is just as wrong to fail to help someone as it is to actively harm them yourself.

It is morally wrong to keep money that one doesn’t really need if one can donate it to causes that provide effective help to those who will benefit a great deal.

It is morally right to harm an innocent person if harming them is a necessary means to helping several other innocent people.

If the only way to ensure the overall well-being and happiness of the people is through the use of political oppression for a short, limited period, then political oppression should be used.

It is permissible to torture an innocent person if this would be necessary to provide information to prevent a bomb going off that would kill hundreds of people.

Sometimes it is morally necessary for innocent people to die as collateral damage—if more people are saved overall.

End of Block: Block 8: Utilitarian scale

Start of Block: Block 9: Demographics

What is your age?

- 18 - 24
- 25 - 34
- 35 - 44
- 45 - 54
- 55 - 64
- 65 - 74
- 75 - 84
- 85 or older
- Prefer not to say

What gender do you identify as?

- Male
- Female
- Non-binary / third gender
- Prefer not to say

What is the highest degree or level of school you have completed? If currently enrolled, highest degree received.

- Primary school
- Some high school
- High school/college graduate, diploma or equivalent
- Trade/technical/vocational training
- Associate degree
- Bachelor's degree
- Master's degree
- Doctorate degree
- Prefer not to say

Which of the following best describes your current employment status?

- Employed full time
- Employed part time
- Unemployed looking for work
- Unemployed not looking for work
- Retired
- Student
- Prefer not to say

What is your ethnic group?

- White
- Black/African/Caribbean/Black British
- Asian/Asian British
- Mixed or multiple ethnic groups
- Other
- Prefer not to say

What is your religion?

- No religion
- Christian (including Church of England, Catholic, Protestant and all other Christian denominations)
- Buddhist
- Hindu
- Jewish
- Muslim
- Sikh
- Other
- Prefer not to say

End of Block: Block 9: Demographics
