# Supplementary material for: Should AI allocate livers for transplant? Public attitudes and ethical considerations
Source: BMC Med Ethics. 2023 Nov 27;24:102. doi: 10.1186/s12910-023-00983-0 (PMC10683249; doi:10.1186/s12910-023-00983-0)
Supplement: Supplementary file 2 — Supplementary Material 2 [file 12910_2023_983_MOESM2_ESM.pdf]

APPENDIX B

Participant demographics

Characteristic	Breakdown	# of participants	% of participants
**Age**	18-24	22	12.8%
	25-34	54	31.4%
	35-44	43	25.0%
	45-54	28	16.3%
	55-64	18	10.5%
	65-74	5	2.9%
	75-84	2	1.2%
	85 or older	0	0.0%
	Prefer not to say	0	0.0%
**Gender**	Male	82	47.7%
	Female	87	50.6%
	Non-binary/third gender	3	1.7%
	Prefer not to say	0	0.0%
**Highest education level**	Primary school	1	0.6%
	Some high school	10	5.8%
	High school/college graduate, diploma or equivalent	51	29.7%
	Trade/technical/vocational training	17	9.9%
	Associate degree	3	1.7%
	Bachelor's degree	66	38.4%
	Master's degree	19	11.0%
	Doctorate degree	5	2.9%
	Prefer not to say	0	0.0%

Characteristic	Breakdown	# of participants	% of participants
**Employment status**	Employed full time	75	43.6%
	Employed part time	33	19.2%
	Unemployed looking for work	14	8.1%
	Unemployed not looking for work	16	9.3%
	Retired	14	8.1%
	Student	13	7.6%
	Prefer not to say	7	4.1%
**Ethnicity**	White	152	88.4%
	Black/African/Caribbean/Black British	4	2.3%
	Asian/Asian British	10	5.8%
	Mixed or multiple ethnic groups	5	2.9%
	Other	0	0.0%
	Prefer not to say	1	0.6%
**Religion**	No religion	108	62.8%
	Christian (including Church of England, Catholic, Protestant and all other Christian denominations)	51	29.7%
	Buddhist	1	0.6%
	Hindu	2	1.2%
	Jewish	0	0.0%
	Muslim	2	1.2%
	Sikh	0	0.0%
	Other	4	2.3%
	Prefer not to say	4	2.3%
