# Supplementary material for: Should AI allocate livers for transplant? Public attitudes and ethical considerations
Source: BMC Med Ethics. 2023 Nov 27;24:102. doi: 10.1186/s12910-023-00983-0 (PMC10683249; doi:10.1186/s12910-023-00983-0)
Supplement: Supplementary file 3 — Supplementary Material 3 [file 12910_2023_983_MOESM3_ESM.pdf]

APPENDIX C

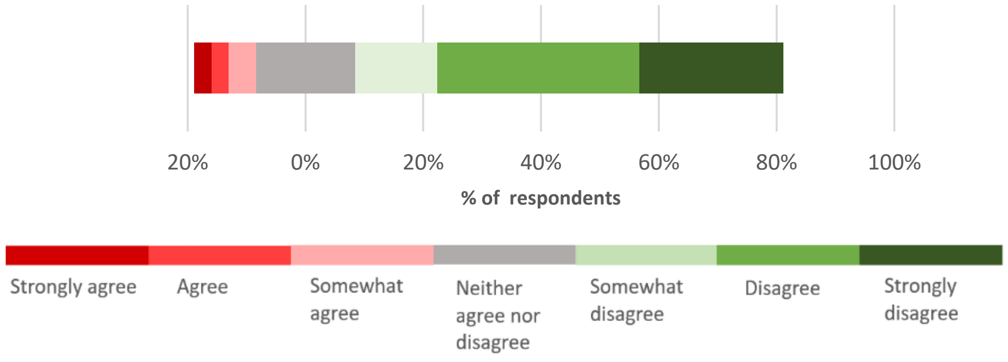

"If AI were used in liver allocation, I would be less likely to donate my organs". Bar represents agreement with the statement.
