# Supplementary material for: Should AI allocate livers for transplant? Public attitudes and ethical considerations
Source: BMC Med Ethics. 2023 Nov 27;24:102. doi: 10.1186/s12910-023-00983-0 (PMC10683249; doi:10.1186/s12910-023-00983-0)
Supplement: Supplementary file 4 — Supplementary Material 4 [file 12910_2023_983_MOESM4_ESM.pdf]

APPENDIX D

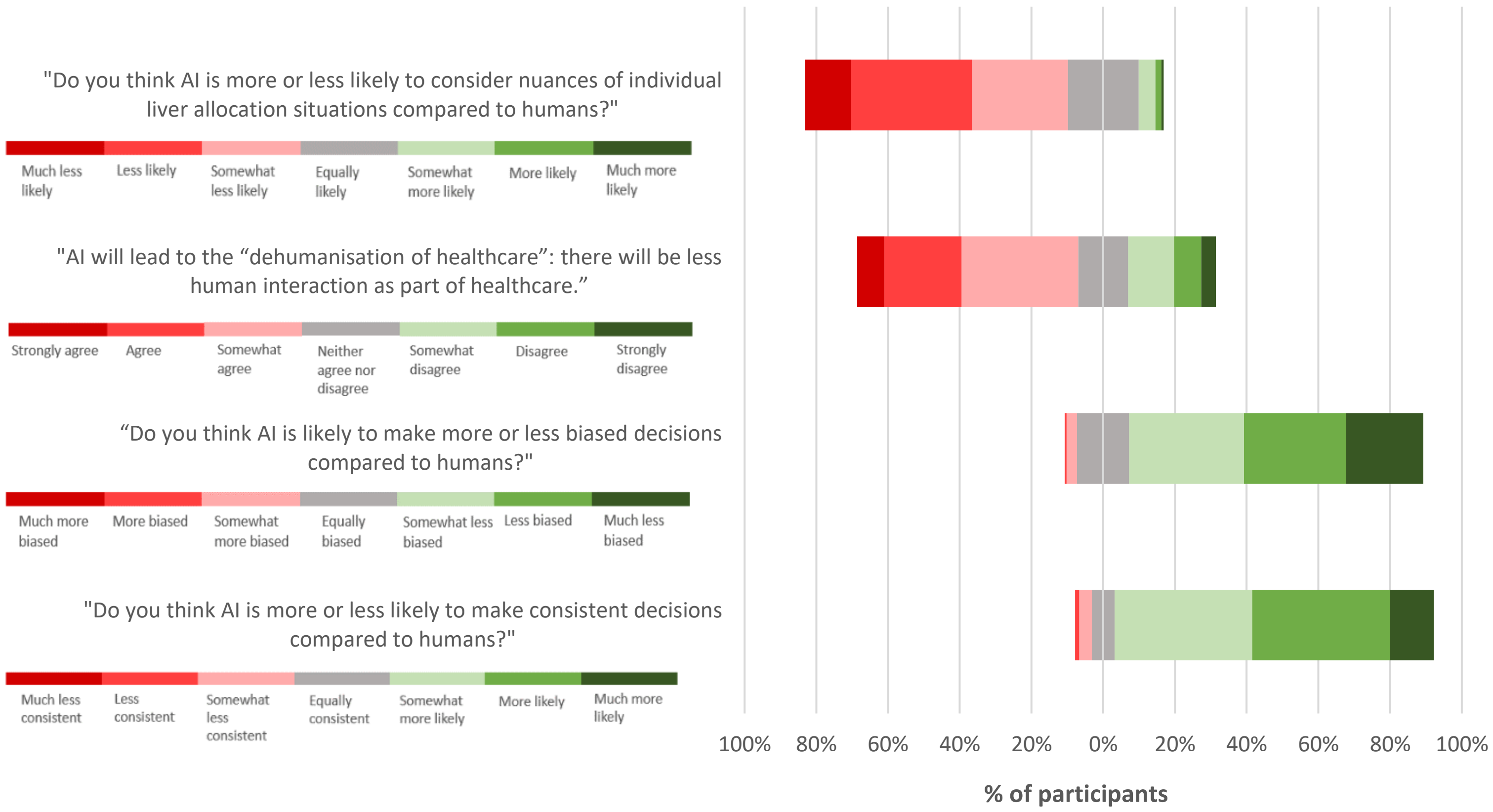

Public attitudes towards AI and nuances of decisions, bias, dehumanisation of healthcare, consistency. Bars represent responses to each question/statement. Each scale is different but is depicted as: not in favour of AI on the left (red) and in favour of AI on the right (green).

There was a slight inconsistency in the scale for the consistency question, which is reflected in the figure.

Further analysis

We used a second exploratory model that included participants' responses to the Oxford Utilitarian Scale (OUS)[41] in addition to ethical attitudes. OUS scores did significantly improve the fit of the first model (Fchange(2, 165) = 4.72, p = .01. The model shows that ethical attitudes significantly predict acceptability ratings, $F(6, 165) = 7.12$, $p < .001$. Ethical attitudes explained approximately 18% of the variation in acceptability ratings (R2adj=.176).

Results showed that acceptability ratings were predicted by the extent to which AI is not perceived as leading to the dehumanisation of healthcare (β =.292, p <.001) and the extent to which AI was perceived as more likely to consider individual nuances (β =-.147, p =.042). Acceptability ratings were also predicted by scores on the Instrumental Harm subscale of the OUS (β = -.208, p =.004): scoring lower on endorsing Instrumental Harm predicted finding AI for liver allocation more unacceptable. Acceptability ratings were not significantly predicted by the extent to which AI is perceived to be more or less biased than humans (β = .127, p =.080), perceived consistency of AI, (β =-.108, p =.138), or Impartial Beneficence scores (β=.066, p=.352).

Another finding that was not included due to space constraints, was that most participants thought that a transplant committee or hospital should be held responsible for a decision in which AI is involved in (Figure 8).

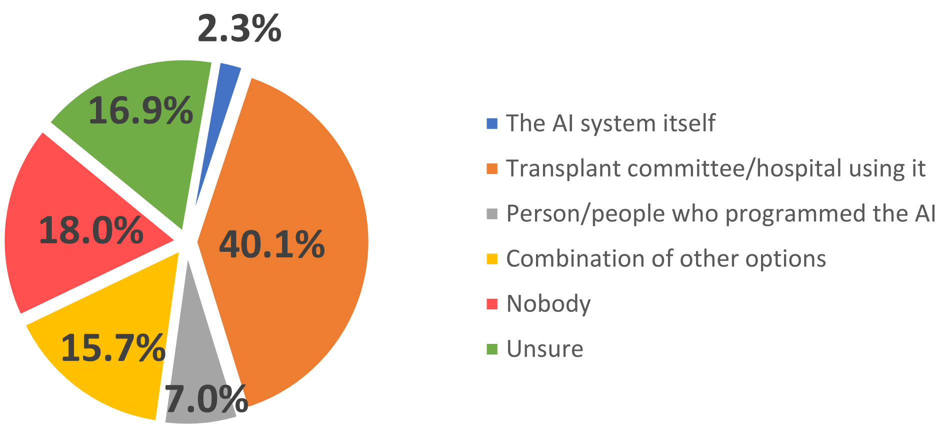

Figure 1: "Who should be held responsible for decisions made by an AI in liver allocation?" Pie chart segments represent responses to the question.

Furthermore, participant attitudes towards AI making predictions of biological outcomes and behaviour were assessed but will be discussed in a further manuscript.
