# Supplementary material for: Should AI allocate livers for transplant? Public attitudes and ethical considerations
Source: BMC Med Ethics. 2023 Nov 27;24:102. doi: 10.1186/s12910-023-00983-0 (PMC10683249; doi:10.1186/s12910-023-00983-0)
Supplement: Supplementary file 5 — Supplementary Material 5 [file 12910_2023_983_MOESM5_ESM.pdf]

APPENDIX E

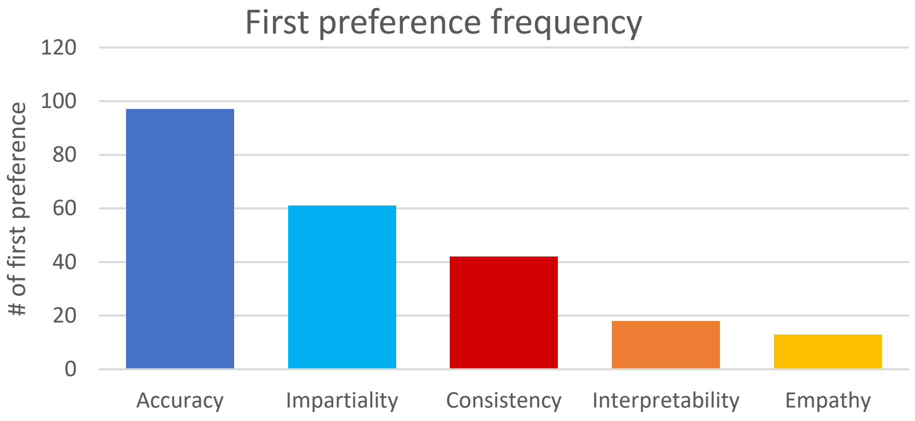

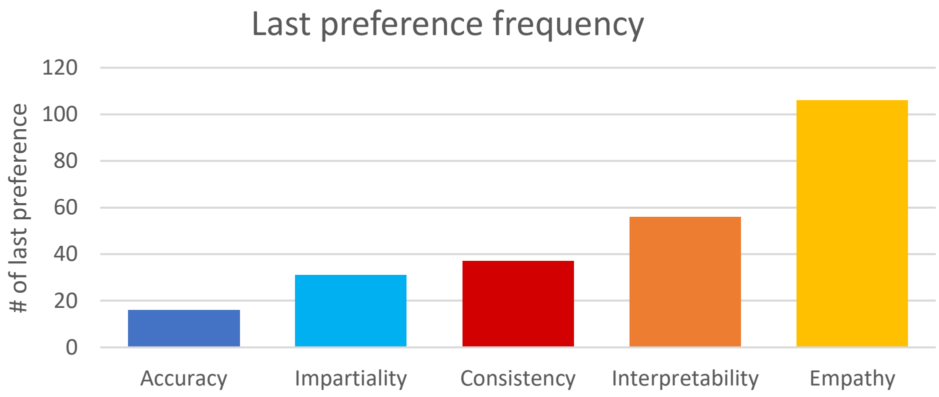

Public attitudes towards the importance of characteristics of decision-makers. The first graph depicts how many participants chose each characteristic as their first preference (including equal firsts). The second depicts how many participants chose each characteristic as their last preference (including equal lasts).

Interpretability: *the decision-maker is able to explain why the allocation decision was made.*
Empathy: *the decision-maker looks at each person as an individual and considers their feelings.*
Accuracy: *the decision-maker makes relevant predictions accurately and weighs up the relevant factors accurately.*
Consistency: *the decision-maker makes decisions based on the same factors every time.*
Impartiality: *the decision-maker is not influenced by outside pressures e.g., money, pressure from the media etc.*
