# Supplementary material for: Should AI allocate livers for transplant? Public attitudes and ethical considerations
Source: BMC Med Ethics. 2023 Nov 27;24:102. doi: 10.1186/s12910-023-00983-0 (PMC10683249; doi:10.1186/s12910-023-00983-0)
Supplement: Supplementary file 6 — Supplementary Material 6 [file 12910_2023_983_MOESM6_ESM.pdf]

APPENDIX F

Participants tended to express greater prioritisation for higher chance of survival in the transplant committee condition ($t=2.96$, $p=.003$).

Participants tended to express more prioritisation for those from a disadvantaged background in the transplant committee condition ($t=2.00$, $p=.048$), although on average this factor was not considered relevant. It is unclear why these differences exist, it appears possible (particularly for the disadvantaged condition) that this may indicate a type 1 error.

Prioritisation judgments did not differ between conditions for any other factors.
